# Genomic revolution of US weedy rice in response to 21st century agricultural technologies

**DOI:** 10.1038/s42003-022-03803-0

**Published:** 2022-09-08

**Authors:** Marshall J. Wedger, Nilda Roma-Burgos, Kenneth M. Olsen

**Affiliations:** 1 Department of Biology, Washington University in St. Louis, St. Louis, MO USA; 2 Department of Crop, Soil and Environmental Sciences, University of Arkansas, Fayetteville, AR USA

**Keywords:** Molecular evolution, Agricultural genetics

## Abstract

Weedy rice is a close relative of cultivated rice that devastates rice productivity worldwide. In the southern United States, two distinct strains have been historically predominant, but the 21^st^ century introduction of hybrid rice and herbicide resistant rice technologies has dramatically altered the weedy rice selective landscape. Here, we use whole-genome sequences of 48 contemporary weedy rice accessions to investigate the genomic consequences of crop-weed hybridization and selection for herbicide resistance. We find that population dynamics have shifted such that most contemporary weeds are now crop-weed hybrid derivatives, and that their genomes have subsequently evolved to be more like their weedy ancestors. Haplotype analysis reveals extensive adaptive introgression of cultivated alleles at the resistance gene *ALS*, but also uncovers evidence for convergent molecular evolution in accessions with no signs of hybrid origin. The results of this study suggest a new era of weedy rice evolution in the United States.

## Abstract (web summary)

Whole-genome re-sequencing of weedy rice accessions reveals recent genetic introgression and adaptation that is altering crop-weed dynamics across southern USA.

## Introduction

Understanding the genomic basis of adaptation is among the most important questions in modern evolutionary biology. Crop domestication has long been recognized as a model for studying adaptive responses to selection^1^, and important insights in the last two decades have come from studies of evolving crop species’ genomes^2,3^. Recently, the evolution of agricultural weeds—which, unlike crops, evolve without intentional selection by humans—are providing additional new insights into the genomics of adaptation^4–6^. Among agricultural weeds, those that are closely related to crop species can be a particularly dynamic system because of the added potential for genetic exchange with crop cultivars as a means of weed evolution and adaptation^7^.

The genomically best-characterized weedy crop relative is weedy rice (*Oryza* spp.), a de-domesticated form of cultivated rice (*O. sativa*)^4,8,9^ that has evolved multiple times independently around the world^4,10,11^. As a feral crop derivative, it is highly adapted and specialized to rice fields, where it competes aggressively with the crop. Just one weedy rice plant per square meter can lead to a >200 kg ha^−1^ loss of yield^12^ and reductions in harvest quality that compromise market value^13–15^. Due to its close phenotypic and genetic similarity to the crop, weedy rice is challenging to control with herbicides and often requires additional specialized field maintenance practices. As a result, weedy rice causes annual economic losses of more than $45 million in the United States (US)^16^ and hundreds of millions of dollars worldwide^17^.

Weedy rice strains worldwide are characterized by a few key shared weed-adaptive features, including a strong seed dispersal mechanism (shattering)^18,19^ and persistent seed dormancy^20^. In the southern US, two phenotypically and genetically distinct morphotypes have historically predominated; strawhull (SH) weedy rice is descended from *indica* rice varieties grown in Asia, while blackhull-awned (BHA) is derived from genetically distinct *aus* Asian varietal group^8,21,22^. Outcrossing rates between SH, BHA, and local US cultivars (all *tropical japonica* varieties) have historically all been <1% despite their close physical proximity within US rice fields^4,23^.

In 2002, non-transgenic herbicide resistant (HR) rice cultivars (marketed as Clearfield^TM^ rice) were first commercialized in the US as a means of controlling weedy rice and other agricultural weeds. These HR cultivars are resistant to the imidazolinone (IMI) class of herbicides due to one of two amino acid replacements in the acetolactate synthase enzyme (ALS). The first Clearfield^TM^ cultivars, CL121 and CL141, carried a G_654_E replacement^24^; they were quickly replaced in 2003 by CL161 and later cultivars, which instead carry an adjacent S_653_N replacement conferring greater herbicide resistance. US HR rice cultivation peaked at ~65% by the mid-2010s and now constitutes ~35% of rice acreage^25^.

Concurrent with the introduction of HR rice, US rice agriculture was further altered by the adoption of hybrid rice technology in place of traditional inbred cultivars. First commercialized in the US in 2000 and now comprising ~50% of US rice acreage (including many HR cultivars)^25^, hybrid rice offers the substantial advantage of enhanced yield through heterosis^26^. However, an unintended consequence of this technology has been the large increase of instances of volunteering^27^, whereby cultivar seeds shatter in the field, overwinter and emerge in subsequent years. Allelic segregation in these hybrid-derived crop volunteers results in a wide range of phenotypic variation, including for flowering time, which increases outcrossing rates with weedy rice^26^. Volunteer rice thus has the potential to serve as a gene-flow bridge, allowing for the escape of HR, and other crop-derived alleles into weedy rice.

The combined adoption of HR and hybrid rice in US agriculture has thus created a two decades long natural experiment: two genetically distinct strains of a historically self-fertilizing weedy crop relative have now been subject to strong selection for herbicide resistance, and this selective pressure has coincided with increased opportunities for crop-weed hybridization via crop volunteers. Notably, as early as 2004, farmers utilizing the Clearfield^TM^ technology reported instances of HR weedy rice^24^. By 2010, 80% of weedy rice plants sampled in one study were classified as resistant and carrying the S_653_N allele derived from HR cultivars^28^. In the decade of continued HR cultivar use that has followed, it is unclear how weedy rice has continued to evolve and adapt, or the extent to which crop × weed hybridization has continued to shape the genetic composition of US weedy rice populations.

In this study we used whole-genome resequencing to investigate how the genomic composition of southern US weedy rice has changed since the 21st century introduction of HR and hybrid rice cultivars. We addressed the following specific questions: 1) How do the genomes of contemporary weeds differ from the historic SH and BHA strains that predominated through the 20th century? 2) Following crop-weed hybridization (creating a weed with 50:50 crop-weed genomic composition), does selection over subsequent generations in weed populations lead to a genome-wide bias toward one ancestral genome or the other? And 3) Within the weed genome, does selection drive known weed- or crop-specific alleles to high frequency in a predictable pattern based on expected advantageous traits for contemporary weeds? Our findings reveal a genomic revolution in US weedy rice in the last 20 years that has irrevocably altered crop-weed dynamics and mechanisms of weed adaptation.

## Results

### Population genetics of contemporary US weedy rice

Seeds from 48 maternal samples across 5 Arkansas rice fields were collected during the harvest season of 2018. US weedy rice lacks geographical genetic structure^4,29^, so this sampling may be considered representative of the southern US rice production region^4,22,29^. Whole-genome sequences (>40× average coverage) were generated using leaf tissue from one seed per maternal plant grown to the seedling stage. Genome assemblies were analyzed with 98 previously published weedy, cultivated, and wild rice samples^4,30–33^ resulting in a dataset of 146 samples and ~19.34 million SNPs. Previously published genomes included 22 historic weedy (11 SH and 11 BHA), 49 cultivated (10 *aus*, 5 *aromatic*, 12 *indica*, 12 *temperate japonica*, and 10 *tropical japonica*) and 27 wild rice accessions. Wild rice accessions were removed from analysis after they were confirmed to play no role in US weedy rice evolution, as was expected given their absence from the US agroecosystem (Supplementary Fig. 1).

**Fig. 1:**
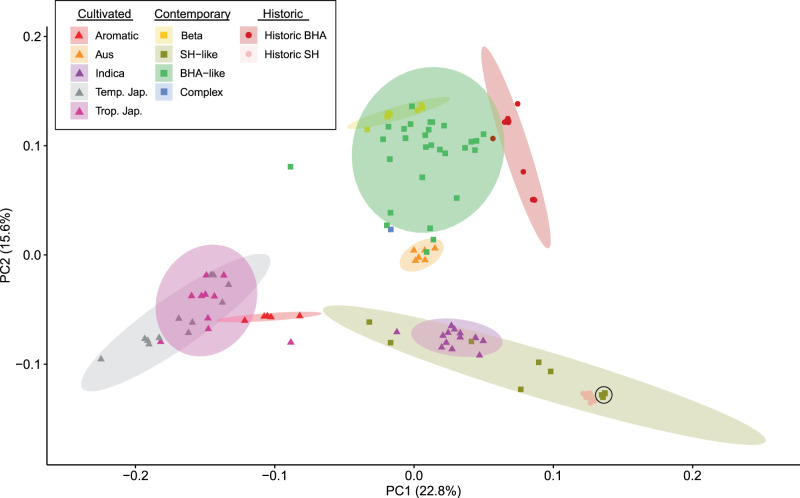
Principal component analysis (PCA) of genome-wide SNPs in weedy and cultivated rice. PCA of genome-wide SNPs in cultivated (*n* = 49), historical weedy (*n* = 22), and contemporary weedy rice (*n* = 48). The first component seperates the *japonica* groups on the left and the *indica* groups on the right. The second component separates SH and SH-like weeds at the bottom from BHA and BHA-like weeds at the top. All hybrid weeds fall between their presumed crop and weed ancestor, consistent with a hybrid origin. Shaded regions represent 95% confidence interval of placement of a theoretical new sample. Black circled outline indicates the position of contemporary SH-like weeds that carry herbicide resistance mutations but have no genome-wide evidence of crop-weed hybrid ancestry.

To assess the overall genetic composition of contemporary weed samples in comparison to historic US weed strains, we employed principal component analysis (PCA) and ADMIXTURE analysis. The PCA revealed relatively tight within-strain grouping of cultivated and historic (pre-2000) weedy rice, with contemporary weedy rice showing a much broader dispersion (Fig. 1). PC1 (22.8% variation explained) separated the *japonica* and *indica* subspecies lineages, which is the deepest divergence in the Asian rice taxonomy. PC2 (15.6% variation explained) separated subgroups within the *indica* subspecies, with *aus* crop varieties and *aus*-like BHA weeds distinguished from *indica* and *indica*-like SH weed samples. Aside from four contemporary weed accessions that cluster very closely with historic SH strains, all other contemporary weeds have intermediate distributions along PC1 between historical weedy rice (SH, BHA) and the US cultivated rice group (*tropical japonica*) (Fig. 1). This suggests that all but four of the contemporary US weed samples are derived from crop-weed hybridization. Among these hybrid descendants, far more appear to be related to historic BHA strains (38 accessions) than to historic SH strains (6 accessions).

**Fig. 2:**
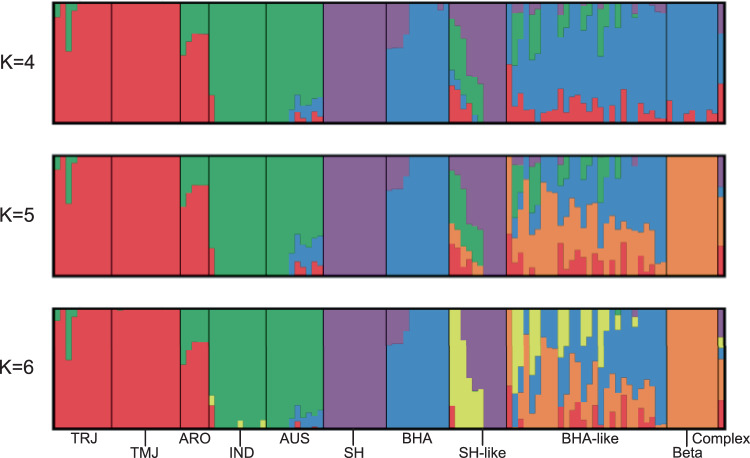
*ADMIXTURE* analysis of contemporary weedy rice in the context of historical weedy and cultivated rice. Results of *ADMIXTURE* analysis of contemporary weedy rice (*n* = 48) (SH-like, BHA-like, Beta, Complex) in comparison to historic weedy rice (SH (*n* = 11), BHA (*n* = 11)) and cultivated rice (TRJ, *tropical japonica* (*n* = 10); TMJ, *temperate japonicaI* (*n* = 12); ARO, *aromatic* (*n* = 5); IND, *indica* (*n* = 12); AUS*, aus* (*n* = 10)). Values of K at 4, 5, and 6 are shown; *K* = 6 is the optimal value based on cross-validation error. Categories for contemporary weeds are based on predominant weedy ancestry. The four solid bars within the SH-like group represent the contemporary weeds without genome-wide evidence of hybridization.

For ADMIXTURE analyses of population structure, CV scores indicated *K* = 6 as the optimal number of populations. However, we believe that *K* = 5 makes the most biological sense since at *K* = 6 and above, the contemporary weeds are subdivided into genetically bottlenecked subgroups, revealing no further information with respect to ancestry (Fig. 2). At *K* = 5, the genetic groups corresponded broadly to the following: *japonica* cultivated varieties (including US cultivars), *indica* cultivated varieties, historic SH weeds, historic BHA weeds, and a genetically homogeneous subgroup within the contemporary weeds that in the PCA are grouped with other crop-BHA hybrid descendants. This genetically homogeneous subset of BHA-like weeds may represent a derivative population of BHA × *tropical japonica* hybrids that emerged early enough after HR cultivar introduction to have evolved into a genetically homogeneous subgroup through multiple generations of inbreeding (see also genetic diversity quantifications below); it is designated the ‘beta’ group in reference to this inferred early origin.

Consistent with results from the PCA, ADMIXTURE analysis suggests that most contemporary US weeds are genetic admixtures descended from hybridization between the historic weed strains and US cultivated rice. At *K* = 5, 35 of 48 contemporary weed accessions (72.9%) had membership assignment coefficients of >15% in two or more genetic populations. Most of these admixed weeds (28 of 48, or 58.3%) appear to be derived from BHA rather than SH historic weeds, which account for 6 of the 48 admixed accessions (12.5%). A single contemporary accession appears to have complex SH-BHA admixed ancestry, with >20% membership coefficients from SH, BHA, and *tropical japonica* genetic populations. Nine samples (18.8%) fell into the homogeneous beta group. As in the PCA, the remaining four contemporary samples (8.3%) were genetically indistinguishable from historical SH weeds. Thus, crop-weed hybridization appears to have given rise to most contemporary US weedy rice, with most of these hybrid derivatives descended from BHA-crop hybridization.

Genetic diversity measures were calculated at every SNP across the genome in order to gain a snapshot of the contemporary weedy rice genome. These measures allowed us to quantify the relative endurance of weed and crop ancestor genomes on a genome-wide scale, and to gauge the relative timing of emergence of the homogeneous beta weed population in comparison to the more heterogeneous contemporary weed groups. Heterozygous SNP quantification indicated that contemporary weeds collectively have a high number of heterozygous sites when compared to their crop ancestors (Supplementary Fig. 2); this is consistent with their relatively recent hybrid ancestry. Among the contemporary hybrid-derived weeds, SH-like weeds averaged higher heterozygosity than BHA-like weeds, with the ‘beta’ subpopulation having significantly lower heterozygosity overall. (Supplementary Fig. 3). In the samples with clear weed and crop admixed ancestry (excluding the ‘complex’ accession), heterozygosity-based estimates of generations since hybridization suggest that most of our samples are five or more generations post-hybridization, with only eight samples less than three generations post-hybridization (Supplementary Fig. 4); these may be conservative estimates, as they assume a return to complete selfing after a single outcrossed generation. Accounting for the soil seed bank and seed dormancy, these results are thus in line with a 20-year-old phenomenon for HR weedy rice evolution via crop-weed hybridization.

### Genome-wide local ancestry

The *Loter* software package^34^ was used to calculate estimates of local ancestry throughout the contemporary weedy rice genome in order to reveal any bias towards crop or weed ancestry that has arisen since hybridization. Notably, the contemporary weeds have shifted away from the 50:50 ratio predicted under neutral genetic drift, and instead show an average of 74.1% and 69.2% assignment to the historical weed genome for BHA-like and SH-like groups, respectively (Table 1, Supplementary Fig. 5). The similarity of these values suggests that both of these independently evolved weed lineages are evolving back toward the historic weed genome at a similar rate. Taken together with the heterozygosity measures above, we can conclude, with high certainty, that the descendants of hybridization events that occurred soon after the introduction of HR rice cultivars have persisted and that they show a clear bias, on a genome-wide level, of evolving back towards their weedy ancestor.

**Table 1:** Average genome bias of contemporary weedy rice.

Chromosome	BHA-like	SH-like
chr1	0.652	0.682
chr2	0.796	0.658
chr3	0.793	0.658
chr4	0.775	0.696
chr5	0.773	0.733
chr6	0.609	0.770
chr7	0.796	0.652
chr8	0.704	0.737
chr9	0.767	0.545
chr10	0.766	0.791
chr11	0.649	0.656
chr12	0.810	0.728
Average	0.741	0.692

Average proportions of contemporary genome called by the *Loter* software as derived from the weedy ancestor for BHA-like and SH-like samples across each of the 12 rice chromosomes.

**Fig. 3:**
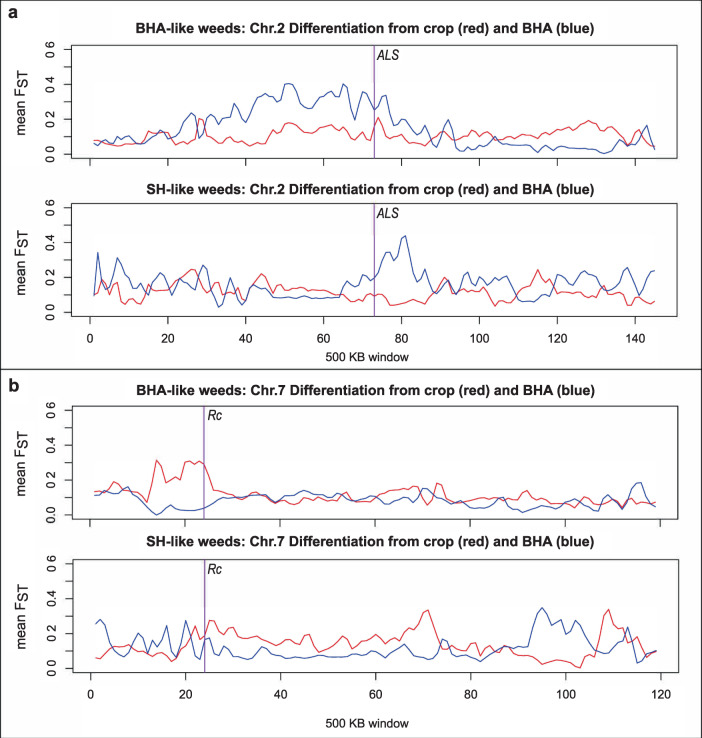
*F*_ST_ analysis across two chromosomes containing genes important for weedy rice survival. *F*_ST_ between contemporary weeds and their presumed ancestors in two chromosomes (chr. 2, **a** and chr. 7, **b**) containing genes associated with contemporary weed adaptation (*ALS*, herbicide resistance; *Rc*, seed dormancy). Red lines represent the *F*_ST_ between cultivated and contemporary weedy populations; blue lines represent *F*_ST_ between historical and contemporary weedy populations. The vertical purple lines denote the 500-kb window containing the focal gene.

**Fig. 4:**
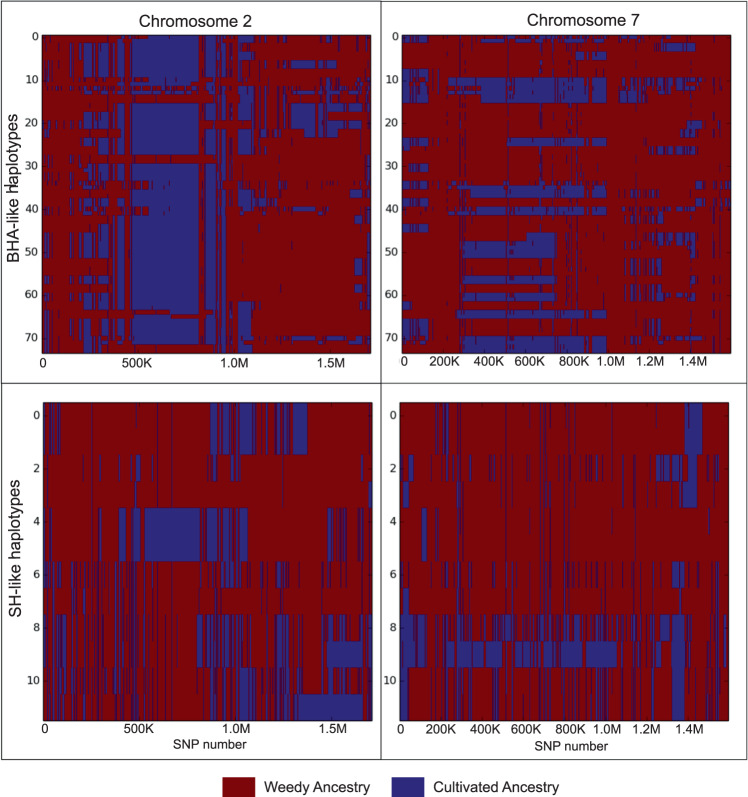
Local ancestry estimations across chromosomes 2 and 7. Local ancestry estimations based on *Loter* analysis across two rice chromosomes (2, 7) for each of two populations of hybrid-derived weedy rice. Each haplotype is plotted horizontally across the relevant chromosome. Blue areas denote crop-like regions of the genome, while red areas represent weed-like regions.

*F*_ST_ was calculated between the hybrid-derived contemporary weeds and their inferred ancestors in a genome-wide sliding window analysis to search for evidence of adaptation via selective introgression of weed or crop alleles. We specifically compared *ALS*, the locus conferring IMI herbicide resistance (where crop alleles are predicted to be strongly favored), with *Rc*, a locus conferring seed dormancy (where weed alleles are predicted to be strongly favored). As hypothesized, we found consistent evidence of a crop-like *ALS* region on chromosome 2 (Fig. 3a). We also identified a weed-like *Rc* region on chromosome 7, although this pattern only held for the BHA-like, and not the SH-like weeds (Fig. 3b). Consistent with the *F*_ST_ sliding window analysis, the *Loter* software identified a large crop-like haplotype block in the region containing *ALS*; interestingly, this was only the case for BHA-like samples (Fig. 4). For *Rc*, *Loter* identified a weed-like region around *Rc*, which could reflect selective maintenance of the dormancy-associated weed allele (or simply the overall genomic shift towards the weed-like genome).

### Haplotype network analysis of ALS

**Fig. 5:**
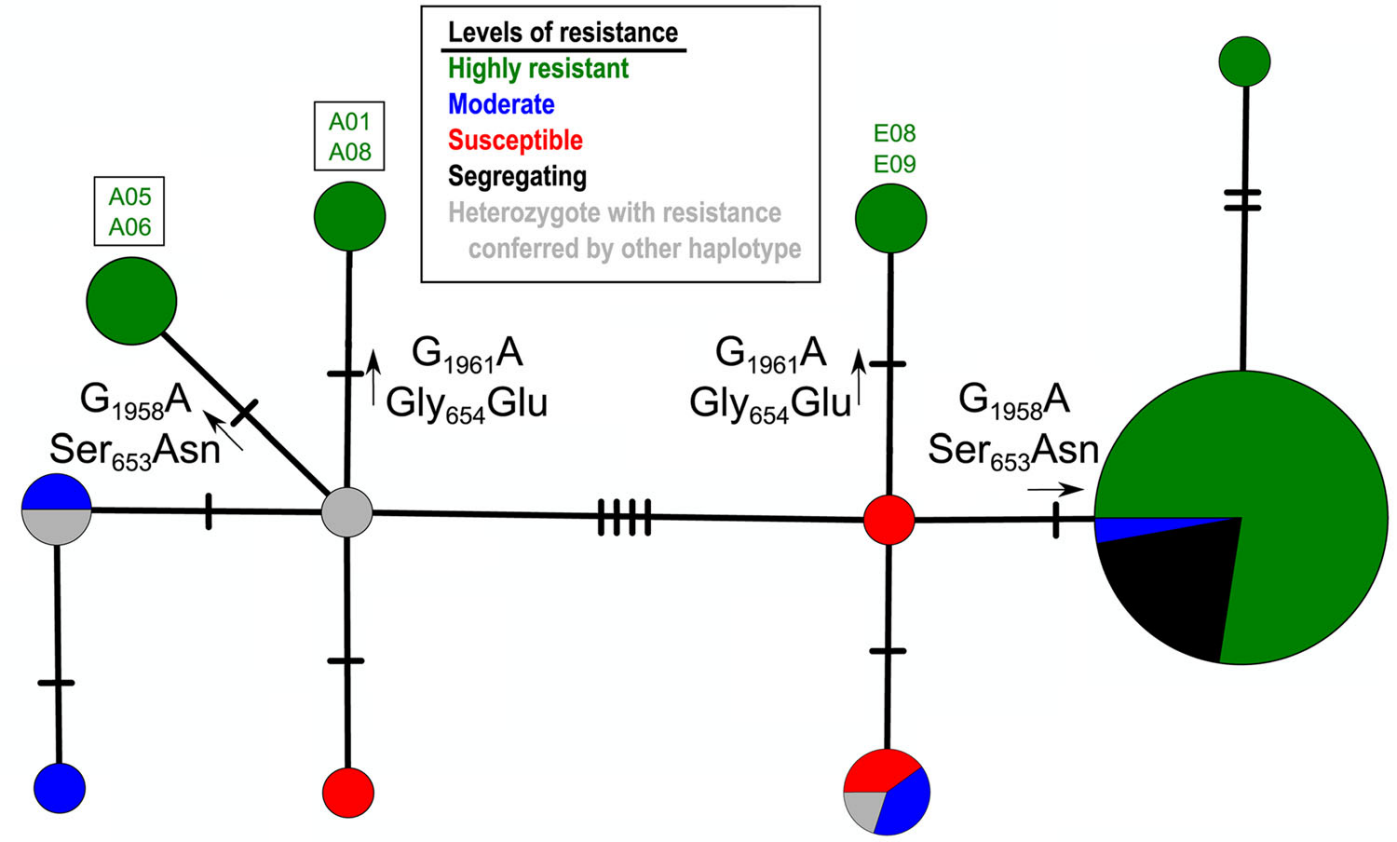
Haplotype tree of the *ALS* herbicide resistance locus. Median joining haplotype tree of the *ALS* herbicide resistance locus from contemporary weedy rice samples. Tree shown is one of four equally parsimonious arrangements (see Supplementary Fig. 6 for alternative topologies). Labeled mutational steps with arrows indicate gain-of-resistance mutations (nucleotide change and corresponding amino acid replacement). Sample names in boxes (A05, A06, A01, A08) are contemporary weed accessions that are not of crop × weed hybrid origin. Sizes of pie chart circles are proportional to haplotype numbers, and colors indicate proportions of herbicide resistance levels.

To gain a finer-scale view of haplotype variation at the *ALS* HR locus, a median joining network tree was constructed from manually phased consensus nucleotide sequences retrieved from assembled raw reads (Fig. 5, Supplementary Fig. 6). The haplotype tree is structured into two diverged haplogroups, with haplotypes on the right side of the network derived from cultivar (*tropical japonica*) ancestry and those on the left side characteristic of weedy ancestry. Most of the contemporary weeds are distributed on the right side of the network and carry the S_653_N mutation and surrounding haplotype sequence present in the widely grown CL161 and later HR cultivars. Two weed samples, E08 and E09, are also on the right side of the network but instead carry the G_654_E mutation and surrounding haplotype indicative of the oldest HR cultivars (CL121 or CL141); this suggests that these two samples are descendants of the very earliest crop × weed hybridization events.

The left section of the haplotype tree, conversely, does not have *ALS* haplotypes of cultivar origin. These haplotypes are represented almost entirely by SH-like plants, consistent with *Loter* results for the *ALS* genomic region. Samples A01 and A08 carry the older G_654_E mutation, but do not show evidence of hybrid origin and occur in a distinctly weed-like haplotype background. This allele was previously shown to have been present in the historical SH population at low frequency^35^, likely due to infrequent exposure to imazethapyr during IMI-resistant soybean rotations. The presence of this allele in contemporary weedy rice populations is thus most likely due to selection on standing variation. Two additional samples, A05 and A06, carry the S_653_N resistance allele, and also show no evidence for hybrid ancestry; this suggests a convergent mutation event conferring resistance. To our knowledge, this is the first report of the S_653_N resistance allele occurring in weedy rice through mutational convergence rather than crop allele introgression. Notably, two common mutations linked to IMI herbicide resistance, Ala122 and Trp574^36^ were not found in the populations examined in this study.

Herbicide resistance phenotyping confirmed that most of our samples showed some level of resistance following application of imazethapyr, with 34/48 samples (70.8%) classified as highly resistant (Table 2). Another 4/48 (8.3%) of samples showed moderate levels of resistance, while 8/48 (16.6%) were segregating for resistance. Thus, the vast majority of contemporary weed genotypes (46/48, or 95.8%) show some degree of herbicide resistance. Only two samples (4.1%) were completely susceptible in our phenotyping trials; both susceptible plants were of crop-weed hybrid origin and were collected from fields not utilizing the Clearfield^TM^ technology. Thus, we suspect they are offspring of parents segregating for resistance. Most plants showing high herbicide resistance carried the common CL161 haplotype (characterized by the S_653_N mutation); additional samples carry the older resistance haplotype of CL121 and CL141 cultivars (characterized by the G_654_E mutation) (Fig. 5). HR phenotyping also confirmed resistance in the four SH weeds that are not of crop-weed hybrid origin and that appear to have evolved resistance through mutational convergence.

**Table 2:** Resistance levels of contemporary weedy rice.

	Field Type			
Resistance	CHY (%)	HYB (%)	CLF (%)	Total (%)
High	14 (93.3)	12 (63.2)	8 (57.1)	34 (70.8)
Moderate	0 (0)	2 (10.5)	2 (14.3)	4 (8.3)
Susceptible	0 (0)	2 (10.5)	0 (0)	2 (4.2)
Segregating	1 (6.6)	3 (15.7)	4 (28.6)	8 (16.7)

Resistance levels of contemporary weedy rice samples. Samples are binned into four categories: high (0–32% average tissue damage), moderate (33–67% average tissue damage), susceptible (68–100% average tissue damage), and segregating. CHY, HYB, and CLF represent field cropping histories representing fields that historically grew Clearfield™ hybrid, non-Clearfield™ hybrid, and Clearfield™ inbred cultivars, respectively.

## Discussion

The results of this study raise a key question: why are the majority of contemporary weedy rice plants of crop-weed hybrid origin? The lack of hybrid persistence prior to 2000^22^ suggests low hybrid fitness. Additionally, the existence of “pre-adapted” HR weedy rice strains, even at initial low frequency, would lead one to expect those fit individuals to quickly rise to high frequency. Instead, reality suggests that those high-fitness individuals only make up ~4% of contemporary samples, while the presumably low-fitness hybrids make up 92% of the contemporary population. One possibility is that while F_1_ fitness is low, fitness of later stages, after selfing, is higher as alleles segregate into favorable configurations.

Local ancestry analysis of BHA-like, “beta”, and SH-like accessions revealed genomes, regardless of ancestry, built primarily of components derived from their weedy rice ancestor. This consistency across weed strains broadly suggests either a selective maintenance of weedy genome components or a selective purge of crop alleles—though these need not be mutually exclusive. The results described here could help inform discussions on crop allele escape (transgenic or otherwise) and the genome-wide process of adaptive introgression in agroecosystems.

The shifting landscape of rice agriculture has resulted in a new generation of weedy rice. The Clearfield^TM^ cropping system has reduced average field infestations drastically, but two decades of herbicide application in the presence of hybrid rice gene-flow bridges has resulted in weedy rice that is herbicide resistant and likely more competitive than historical populations. The rapid adaptation of weedy rice to herbicide application should serve as yet another example of the dangers of relying on single methods of control for agricultural pests.

## Methods

### Plant materials

Contemporary weedy rice plants were collected from rice fields in Greene County, AR, in late August 2018. Fields were selected based on their cropping history as reported by local farmers and are representative of the major rice growing area of the southern US. Previous population genetic studies have documented that the weed strains show no evidence of geographical population structure across the southern US rice growing region^8^. Samples were collected from fields representing three different rice cropping histories: HR inbred cultivars (1 field, 14 plants); HR hybrid cultivars (1 field, 15 plants), and hybrid non-HR cultivars (3 fields, 19 plants) (TABLE S1). Where applicable (43 of 48 samples), full mature panicles were clipped and collected in the field from weedy rice plants no closer than 5 meters from another collection site. For the remaining samples, where seeds had not yet reached maturity, plants were transplanted to Washington University in St. Louis (WUSTL) greenhouse facilities in 18-gallon plastic bins and brought to seed maturity in growth chambers (28 °C, 16:8 h day:night, 60% humidity).

### Whole-genome sequencing

One seed per field-sampled plant was brought to seedling stage, from which fresh leaf tissue was collected and ground in liquid nitrogen for DNA extraction using a modified CTAB protocol^37^. It should be noted that the DNA for this study was collected from plants grown and selfed one additional generation in the greenhouse; therefore, variation observed in genome sequence data may not correspond perfectly to field-collected genotypes, particularly for segregating variants in hybrid derivatives. Illumina libraries were generated in house using a Nextera DNA Flex library prep kit with Nextera DNA CD indexes with the i5 bases recommended for HiSeq 3000/4000 (Illumina, San Diego, CA). Samples were multiplexed following recommendations from Nextera and sent to Novogene (Novogene Corporation Inc., Sacramento, CA) for paired-end short-read sequencing on the HiSeq X 10 platform. Raw reads were de-multiplexed by Novogene before data return.

### Data collection and SNP filtering

Whole-genome sequencing reads from contemporary weedy rice samples were combined with raw reads from previously published whole-genome studies ^30–33^ resulting in a full dataset of 146 samples representing cultivated, weedy and wild rice (Supplementary Data 1). All SNP identification and filtering was performed using the full dataset. Raw reads were trimmed for quality control using default parameters in *Trimmamatic*^38^, followed by alignment to the MSU version 7.0 rice reference genome^39^ using *BWA*^40^. Aligned sequences were sorted and converted to.bam files using *samtools*. The *mpileup* program in the *bcftools* software^41^ was used for variant calling and conversion to the.vcf file type. Finally, *vcftools*^42^ was used to filter out indels, remove variants with a minor allele frequency <0.05, and remove sites clearly out of Hardy–Weinberg equilibrium (*p* < 0.0000001). *Vcftools* was also used to remove wild samples from.vcf files for analyses where they were not required (described below).

### Population genetic analyses

The *pca* flag within *plink*^43^ was used in conjunction with *ADMIXTURE*^44^ to determine population structure (Supplementary Fig. 1). ADMIXTURE results were visualized in *pong*^45^. Wild rice was found to show little-to-no overlap with contemporary US weedy rice and was removed from further analysis. Principal component analysis (PCA) and *ADMIXTURE* analysis showed that grouping contemporary weeds by field type was uninformative for explaining population structure; they were therefore grouped and analyzed based on their predominant weedy rice ancestry in subsequent analyses. From the ADMIXTURE and PCA results combined, contemporary weeds were categorized into three groups: “SH-like”, defined as >10% SH ancestry in ADMIXTURE (without BHA contribution) or placement in the PCA output as intermediate between historic SH and cultivated *tropical japonica* strains; ‘BHA-like’, defined as >10% BHA ancestry in ADMIXTURE (without SH contribution); and the ‘beta’ group, defined based on placement with BHA-like weeds in the PCA (and in the ADMIXTURE analysis at *K* = 4), but with assignment to its own unique genetic population in the ADMIXTURE analysis at *K* ≥ 5. A single contemporary weed accession with complex admixed ancestry was assigned its own category (‘complex’).

Heterozygous sites among genome-wide SNPs were calculated per accession using the *-het* flag in the *plink* software. Wright’s *F*_*ST*_ was calculated for each contemporary weed group in relation to its weed and crop ancestors using the *–weir-fst-pop* flag in the *vcftools* software, with a window size of 500 kb and a 250 kb step size. The first *F*_*ST*_ calculation measured differentiation between a given contemporary weed group (as identified in population structure analyses) and the predominant weed ancestor of that group (SH or BHA), while the second measured differentiation between that weed group and the rice variety group representing US cultivars (*tropical japonica*). These *F*_*ST*_ values were then plotted together across the 12 chromosomes of the rice genome to identify genomic regions with differential contributions of the weed or crop ancestor. Average pairwise nucleotide diversity (π) values for contemporary weed groups were calculated and visualized in the same way using the *–site-pi* flag in the *vcftools* software. As a measure of inbreeding, homozygous locus counts were performed in *vcftools* using the *-het* flag and converted to fraction of heterozygous loci using the formula ((N_sites – O(HOM))/N_sites). Lastly, a custom python script was developed to identify ancestrally-informative SNPs (defined here as sites that are fixed differences between the presumptive ancestors of a hybrid individual). This script then calculated observed heterozygous genotype counts at those sites only. This analysis allowed us to estimate the number of generations since hybridization, assuming a 50% reduction in the number of heterozygous genotypes per generation and a return to a strictly selfing mating system.

### Local ancestry

To complement *F*_*ST*_ analyses, local ancestry across the genome was calculated for weed groups using the *Loter* software^34^ and visualized with *matplotlib*^46^. A custom python script was used to quantify the proportion of ancestral genomes (crop vs. weed) found in the contemporary hybrids. A second custom python script was written to convert an MSU-7.0 genomic location to the corresponding bin of the *Loter* output. This allowed us to pinpoint potential candidate genes for weed adaptation.

### ALS haplotype network analyses

The *samtools*^41^ software package was used to retrieve raw reads mapping to the *ALS* gene region from sorted.bam files. These raw reads were retrieved by using the *index*, *view* (specifying the known gene boundaries) and *fasta* commands. Raw reads were then exported to the *Geneious* 8.1.6 software (https://www.geneious.com) for assembly to a reference *ALS* sequence obtained from GenBank (accession MH636577). After assembly, sequences were trimmed to match the reference sequence and manually phased to remove heterozygous calls from consensus sequences. Phased and trimmed sequences were exported to the *PopART* software^47,48^ for haplotype network visualization.

### Herbicide resistance phenotyping

Weedy rice seeds were planted at 1.27 cm depth into pots (15.24-cm top diameter) filled with 50:50 mixture by volume of field soil and Sunshine potting mix. Up to 12 seeds were planted per pot, depending on the quantity of seeds available per sample. The pots were placed in a greenhouse with supplemental lighting to achieve a 16-h daylength. The temperature was set at a minimum of 25 °C and maximum of 35 °C. At the 3-leaf stage, the plants were treated with 70 g ai ha^−1^ imazethapyr two times, 10 days apart. Imazethapyr was applied in 187 L ha^−1^ spray volume, in a spray chamber with a motorized spray boom fitted with two 800067 flat fan nozzles spaced 46 cm apart. The herbicide treatment was replicated three times and a nontreated check for each sample served as reference for evaluation of plant response. Visible injury was evaluated 3 weeks after the second application of imazethapyr on a scale of 0 to 100% where 0 indicated no injury and 100 indicated a dead plant. The level of injury reflects the level of resistance to imazethapyr.

### Statistics and reproducibility

This study is based on the short-read whole-genome sequencing of 119 weedy and cultivated rice strains, and all population genomic analyses were performed using publicly available software described in the Methods. All statistical analyses were preformed using programs described in the Methods. Reproducibility, including parameters for the population genomic analyses, sample sizes and number of replicates, are stated in Methods and Figures.

### Reporting summary

Further information on research design is available in the Nature Research Reporting Summary linked to this article.

## Supplementary information

Supplementary Information

Description of Additional Supplementary Files

Supplementary Data 1

Reporting Summary

## Data Availability

The new sequence data generated and analyzed in this manuscript are available in the GenBank genetic sequence database. BioProject ID PRJNA847219: accessions SAMN28922700-SAMN28922747.

## Code availability

Custom python scripts written for data analysis can be found at the 10.5281/zenodo.6954804.
