# Supplementary material for: Genomic revolution of US weedy rice in response to 21st century agricultural technologies
Source: Commun Biol. 2022 Sep 8;5:885. doi: 10.1038/s42003-022-03803-0 (PMC9458635; doi:10.1038/s42003-022-03803-0)
Supplement: Supplementary file 1 — Supplementary Information [file 42003_2022_3803_MOESM1_ESM.pdf]

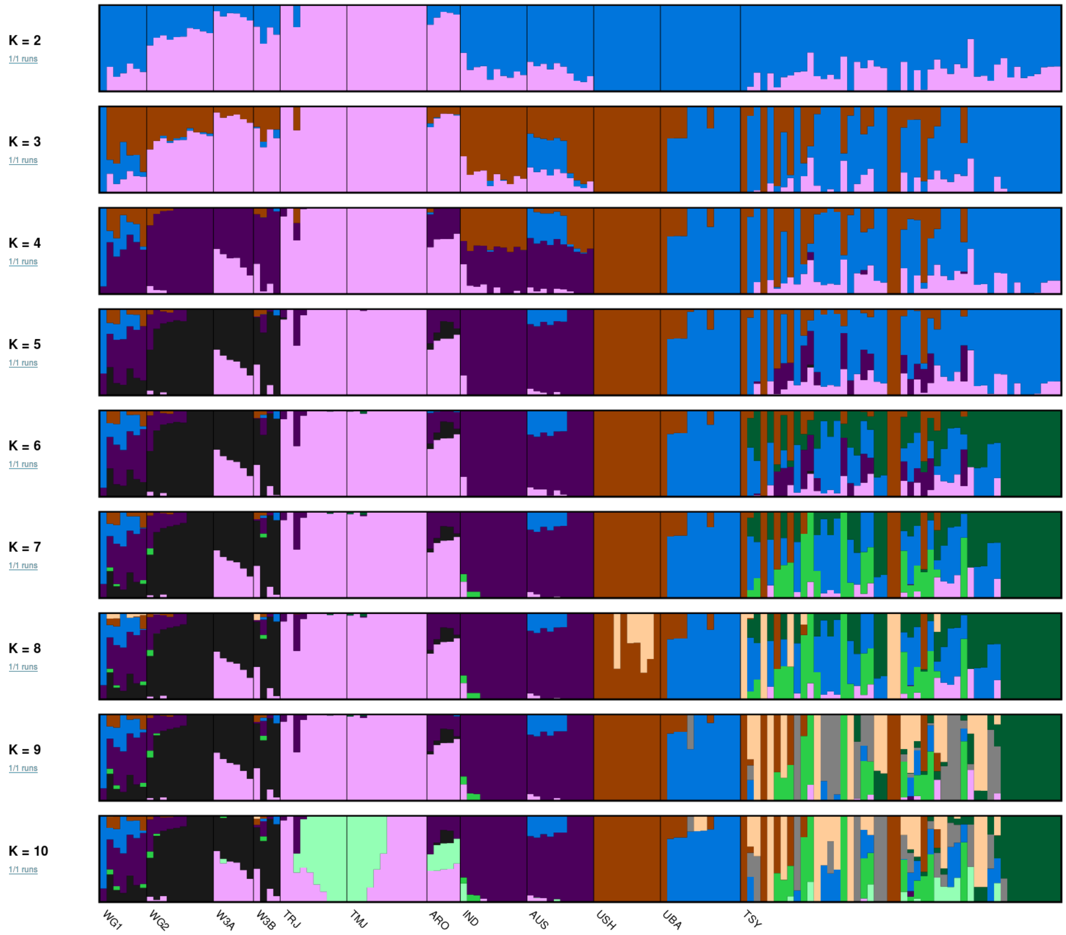

Supplementary Figure 1: *ADMIXTURE* results at K=2-10 for contemporary weeds collected for this study (TSY) in comparison to historic weedy rice (SH, BHA), cultivated rice (TRJ, *tropical japonica*; TMJ *temperate japonica*; ARO, *aromatic*; IND, *indica;* AUS, *aus*), and wild rice (WG1, WG2, W3A, W3B).

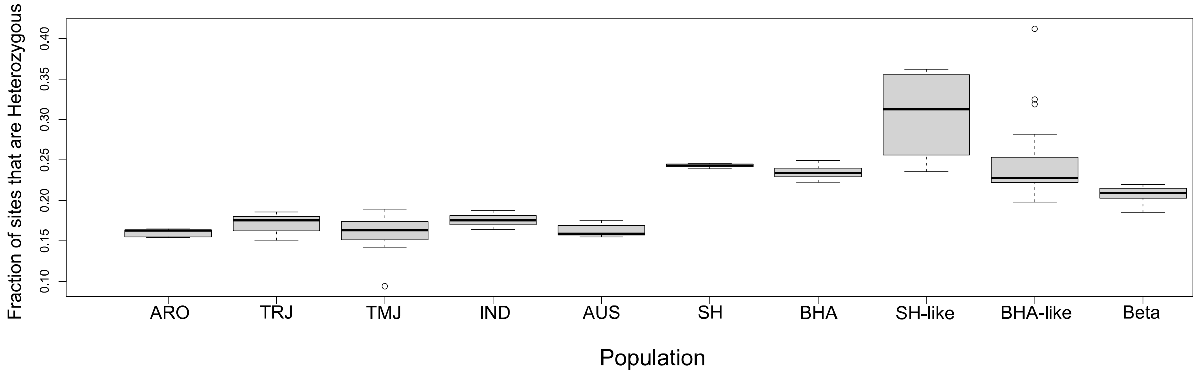

Supplementary Figure 2: Proportion of SNPs that were called as heterozygous among populations of cultivated rice (TRJ, *tropical japonica*; TMJ *temperate japonica*; ARO, *aromatic*; IND, *indica;* AUS*, aus*), historical weedy rice (SH, BHA), and contemporary weedy rice of hybrid origin (excluding the 'complex' sample) (SH-like, BHA-like, and Beta). Box plots show median, inter-, and outerquartile ranges of all samples in the population.

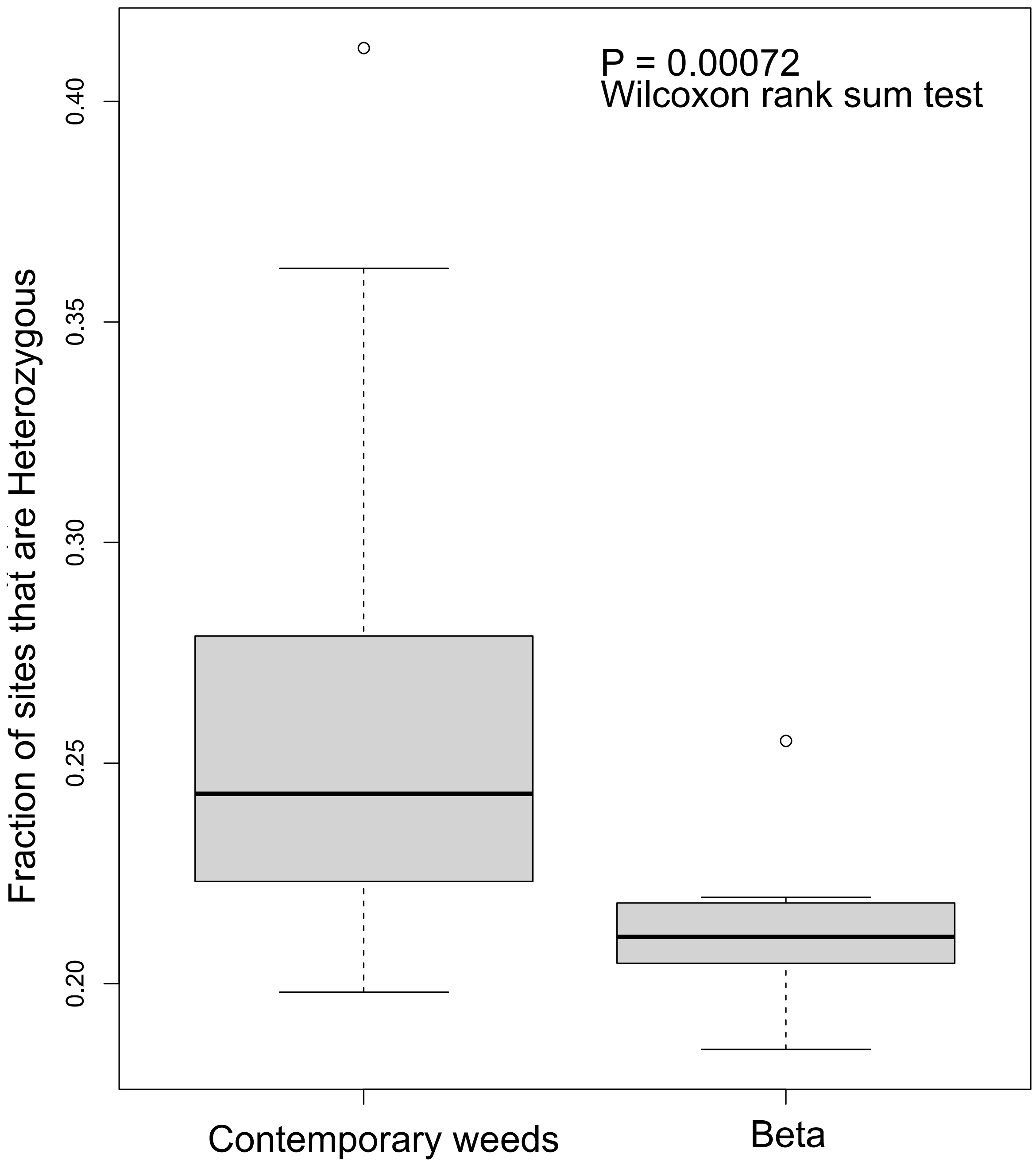

Supplementary Figure 3: Fraction of sites called as heterozygous in Beta population against the rest of the contemporary weeds. Beta weeds have significantly fewer heterozygous sites, which suggests many more generations since the hybridization event.

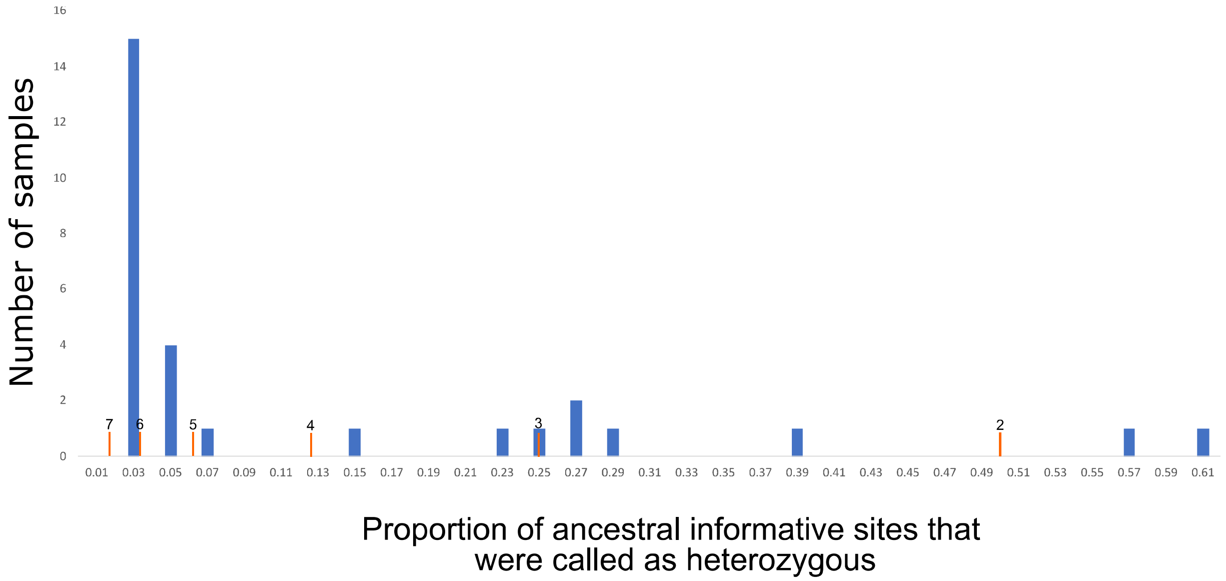

Supplementary Figure 4: Histogram of the proportion of ancestrally informative sites that were called as heterozygous. Thick blue bars represent the number of samples that fell into 0.02-unit wide bins. Thin orange lines represent the expectation of heterozygosity loss per generation with selfing under neutral genetic drift, where 50% of heterozygosity is lost per generation. The number above the thin orange lines represents the expected generation.

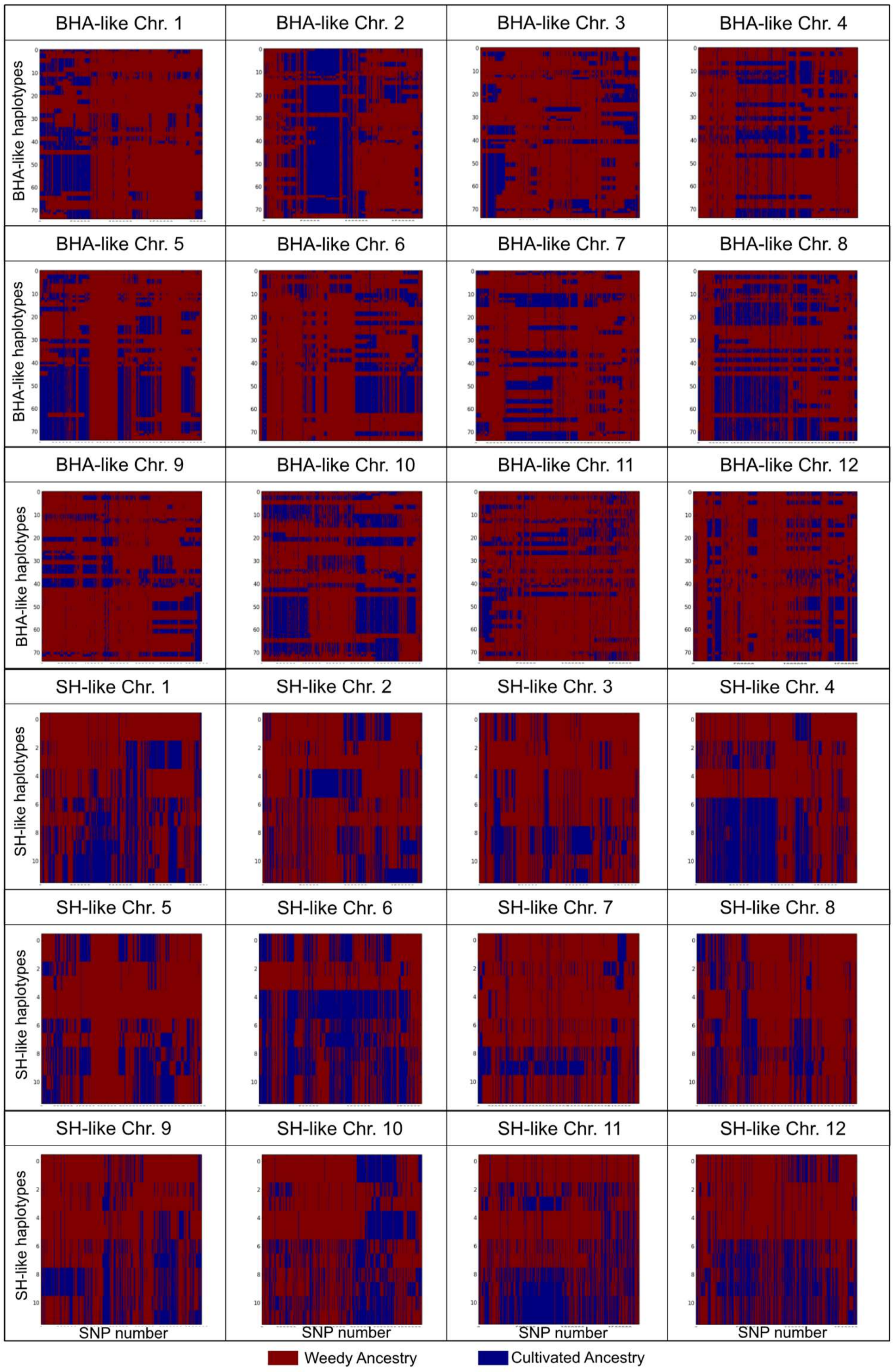

Supplementary Figure 5: *Loter* output for BHA-like and SH-like weeds across all 12 rice chromosomes. Red represents genomic locations derived from ancestral weedy rice, while blue represents ancestry from cultivated rice.

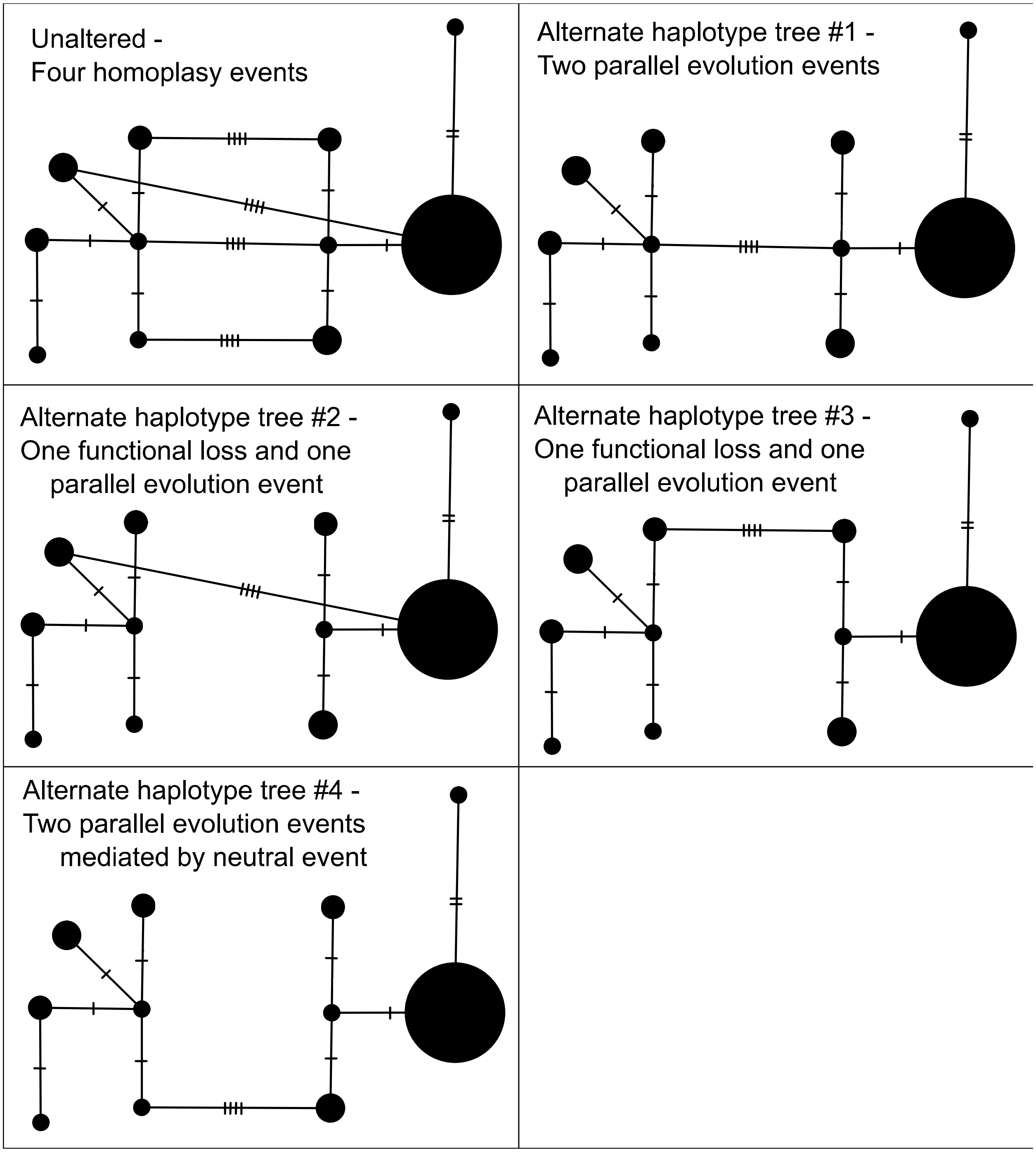

Supplementary Figure 6: All possible median joining haplotype trees for the *ALS* locus including the unaltered tree and four alternative trees with homoplasies removed. Mutational scenarios are provided for each tree.

Supplementary Table 1: List of all samples collected and used in this study. (See online supplementary information.)
