# Supplementary material for: Genomic revolution of US weedy rice in response to 21st century agricultural technologies
Source: Commun Biol. 2022 Sep 8;5:885. doi: 10.1038/s42003-022-03803-0 (PMC9458635; doi:10.1038/s42003-022-03803-0)
Supplement: Supplementary file 2 — Description of Additional Supplementary Files [file 42003_2022_3803_MOESM2_ESM.pdf]

Description of Additional Supplementary Files

File name: Supplementary Data 1
Description: List of samples collected and used in the study.
