# Supplementary material for: Genomic revolution of US weedy rice in response to 21st century agricultural technologies
Source: Commun Biol. 2022 Sep 8;5:885. doi: 10.1038/s42003-022-03803-0 (PMC9458635; doi:10.1038/s42003-022-03803-0)
Supplement: Supplementary file 4 — Reporting Summary [file 42003_2022_3803_MOESM4_ESM.pdf]

nature portfolio

Corresponding author(s): Kenneth M. Olsen

Last updated by author(s): Jul 12, 2022

Reporting Summary

Nature Portfolio wishes to improve the reproducibility of the work that we publish. This form provides structure for consistency and transparency in reporting. For further information on Nature Portfolio policies, see our Editorial Policies and the Editorial Policy Checklist.

Statistics

For all statistical analyses, confirm that the following items are present in the figure legend, table legend, main text, or Methods section.

n/a	Confirmed	
☐	☒	The exact sample size (*n*) for each experimental group/condition, given as a discrete number and unit of measurement
☐	☒	A statement on whether measurements were taken from distinct samples or whether the same sample was measured repeatedly
☐	☒	The statistical test(s) used AND whether they are one- or two-sided<br>*Only common tests should be described solely by name; describe more complex techniques in the Methods section.*
☒	☐	A description of all covariates tested
☒	☐	A description of any assumptions or corrections, such as tests of normality and adjustment for multiple comparisons
☒	☐	A full description of the statistical parameters including central tendency (e.g. means) or other basic estimates (e.g. regression coefficient) AND variation (e.g. standard deviation) or associated estimates of uncertainty (e.g. confidence intervals)
☒	☐	For null hypothesis testing, the test statistic (e.g. *F*, *t*, *r*) with confidence intervals, effect sizes, degrees of freedom and *P* value noted<br>*Give P values as exact values whenever suitable.*
☒	☐	For Bayesian analysis, information on the choice of priors and Markov chain Monte Carlo settings
☒	☐	For hierarchical and complex designs, identification of the appropriate level for tests and full reporting of outcomes
☒	☐	Estimates of effect sizes (e.g. Cohen's *d*, Pearson's *r*), indicating how they were calculated

Our web collection on statistics for biologists contains articles on many of the points above.

Software and code

Policy information about availability of computer code

Data collection	N/A
Data analysis	Data analysis software is extensively covered in Methods portion of the manuscript. Here in brief: SNP filtering was completed using Trimmamatic, BWA, samtools, mpileup, bcftools, and vcftools at their latest version in June 2021. Further data analysis and visualization was done using plink1.9, ADMIXTURE1.3, pong1.4.9, Loter, popArt, and matplotlib3.5.2. Custom python3 scripts were produced to estimate generations since hybridization, streamline Loter runs, and convert MSU-7.0 genomic locations to approximate Loter positions. These scripts can be made available upon request.

For manuscripts utilizing custom algorithms or software that are central to the research but not yet described in published literature, software must be made available to editors and reviewers. We strongly encourage code deposition in a community repository (e.g. GitHub). See the Nature Portfolio guidelines for submitting code & software for further information.

Data

Policy information about availability of data

All manuscripts must include a data availability statement. This statement should provide the following information, where applicable:

- Accession codes, unique identifiers, or web links for publicly available datasets
- A description of any restrictions on data availability
- For clinical datasets or third party data, please ensure that the statement adheres to our policy

The new sequence data generated and analyzed in this manuscript are available in the GenBank genetic sequence database. BioProject ID PRJNA847219: accessions SAMN28922700-SAMN28922747.

Field-specific reporting

Please select the one below that is the best fit for your research. If you are not sure, read the appropriate sections before making your selection.

☐ Life sciences ☐ Behavioural & social sciences ☒ Ecological, evolutionary & environmental sciences

For a reference copy of the document with all sections, see nature.com/documents/nr-reporting-summary-flat.pdf

Ecological, evolutionary & environmental sciences study design

All studies must disclose on these points even when the disclosure is negative.

Study description	Genomic study of 48 contemporary weedy rice samples in the united states to determine the effects of novel rice cultivation practices on associated weeds.
Research sample	48 weedy rice (Oryza sativa) samples collected from Arkansas rice fields. these contemporary samples were compared against historical samples collected from the 1990's to determine the effects of agricultural practices introduced in the early 2000's.
Sampling strategy	Samples were collected from infested rice farms. Samples were >5 meters from previous collections. Fields were chosen based on their cropping history. Fields had at least a decade of growing of either 1) traditional inbred 2) inbred herbicide resistant or 3) hybrid herbicide resistant rice. Data analysis showed these cropping histories had no noticeable effect on population structure. Sample size calculations were not made beforehand, but samples represent a full day of driving and collecting. Seeds were taken when possible, but some immature plants were dug up and transported to the Wash-U greenhouse until maturation where seeds were harvested.
Data collection	Data were recored by Nilda R. Burgos and Marshall J. Wedger.
Timing and spatial scale	Data were collected once in late August 2018.
Data exclusions	No samples were excluded from data analysis. Some SNPs were excluded for standard SNP filtering reasons described in the manuscript.
Reproducibility	Sequencing and data analysis were not repeated.
Randomization	Sequences were pooled after barcoding, but due to the non-experimental nature of this manuscript, randomization did not play a large role.
Blinding	Blinding was not utilized during data analysis. During data collection, however, Nilda R. Burgos is an expert at identifying weedy rice and as such is best suited to fair collections.

Did the study involve field work? ☒ Yes ☐ No

Field work, collection and transport

Field conditions	Collections occurred in late August 2018 when US rice reaches maturation. This specific collection occurred after a heavy rainfall, but we do not suspect it has any relevance to our results.
Location	Collection occurred in Greene county, Arkansas. Our partner farmers have asked us not to publish the GPS coordinates of their fields.
Access & import/export	The USDA rice extension officer in Arkansa, Nilda R. Burgos, and RiceTec kindly allowed us access to their network of professionals who we asked, and received permission from, to enter their rice fields. No permits were required to collect or transport.
Disturbance	N/A

Reporting for specific materials, systems and methods

We require information from authors about some types of materials, experimental systems and methods used in many studies. Here, indicate whether each material, system or method listed is relevant to your study. If you are not sure if a list item applies to your research, read the appropriate section before selecting a response.

Materials & experimental systems

n/a	Involved in the study
☒	☐ Antibodies
☒	☐ Eukaryotic cell lines
☒	☐ Palaeontology and archaeology
☒	☐ Animals and other organisms
☒	☐ Human research participants
☒	☐ Clinical data
☒	☐ Dual use research of concern

Methods

n/a	Involved in the study
☒	☐ ChIP-seq
☒	☐ Flow cytometry
☒	☐ MRI-based neuroimaging
